# Inorganic Thermoelectric Fibers: A Review of Materials, Fabrication Methods, and Applications

**DOI:** 10.3390/s21103437

**Published:** 2021-05-14

**Authors:** Jiwu Xin, Abdul Basit, Sihui Li, Sylvain Danto, Swee Chuan Tjin, Lei Wei

**Affiliations:** 1 School of Electrical and Electronic Engineering, Nanyang Technological University, Singapore 639798, Singapore; D201677257@hust.edu.cn; 2 Department of Physics, National Changhua University of Education, Changhua City 50074, Taiwan; I201622048@hust.edu.cn; 3 State Key Laboratory of Materials Processing and Die and Mould Technology, Huazhong University of Science and Technology, Wuhan 430074, China; D201780274@hust.edu.cn; 4 National Centre for Scientific Research (CNRS), University of Bordeaux, F-33600 Pessac, France; sylvain.danto@u-bordeaux.fr; 5 The Photonics Institute, Nanyang Technological University, 50 Nanyang Avenue, Singapore 639798, Singapore

**Keywords:** thermoelectrics, fibers, inorganic materials, flexible electronics

## Abstract

Thermoelectric technology can directly harvest the waste heat into electricity, which is a promising field of green and sustainable energy. In this aspect, flexible thermoelectrics (FTE) such as wearable fabrics, smart biosensing, and biomedical electronics offer a variety of applications. Since the nanofibers are one of the important constructions of FTE, inorganic thermoelectric fibers are focused on here due to their excellent thermoelectric performance and acceptable flexibility. Additionally, measurement and microstructure characterizations for various thermoelectric fibers (Bi-Sb-Te, Ag_2_Te, PbTe, SnSe and NaCo_2_O_4_) made by different fabrication methods, such as electrospinning, two-step anodization process, solution-phase deposition method, focused ion beam, and self-heated 3ω method, are detailed. This review further illustrates that some techniques, such as thermal drawing method, result in high performance of fiber-based thermoelectric properties, which can emerge in wearable devices and smart electronics in the near future.

## 1. Introduction

Globally, natural energy reservoirs such as coal, oil, and gas have greatly been consumed in industrial power generation with the increasing electricity demand [1]; thus, developing eco-friendly and sustainable energy resources has become an urgent need. Meanwhile, portable electronic devices, such as mobile phones, watches, microcomputers, and health monitoring systems, have achieved rapid development and progress in miniaturization and integration, which greatly promotes the era of intelligent Internet of Things (IOT) [2,3]. In this scenario, thermoelectric (TE) materials have the potential to realize the conversion of waste heat and electricity, which can be used to address the abovementioned challenges [4,5,6]. However, the conversion efficiency of thermoelectric devices is closely related to the *ZT* of TE materials; *ZT* is the dimensionless figure of merit of the materials employed, and *ZT* = *S*^2^*σT*/*κ*, where *σ*, *S*, and *κ* are corresponding to the electrical conductivity, Seebeck coefficient, and thermal conductivity, respectively. *T* is the absolute temperature or the temperature difference between the environment and human skin [7]. So far, achieving a high *ZT* of bulk TE material requires high electrical performance and low thermal conductivity, which is often challenging to effectively decouple the electrical and thermal properties owing to the nature of carrier transports [8].

Flexible thermoelectrics (FTE) have recently gained much attention due to their portability and good compatibility with the IOT family originated by their intrinsic Seebeck, as well as Peltier, effect [9]. FTE are usually composed of natural or synthetic thermoelectric fibers which possess natural advantages such as good mechanical properties and flexibility. Many nanostructured thermoelectrics, including nanowires [10], nanotubes [11], superlattices [12,13], and bulk materials, tend to achieve an extensive phonon scattering at boundaries and interfaces [14,15] which results in inherent flexibility and low thermal conductivity. Currently, fiber-shaped form factors are widely used in engineering technology [16,17,18] to enable various equipment designing [19] or fiber-based thermoelectrics specifically. Portable and wearable thermoelectric materials can be classified into organic fibers, inorganic fibers, and inorganic/organic hybrid fibers, in terms of their compositions [20]. Among the accessible materials, inorganic fibers are superior compared to the organic fibers regarding the heat resistance, stability, and high thermoelectric performance. Herein, we review the development of inorganic thermoelectric devices achieved by Bi_2_(Te, Se)_3_, Ag_2_Te, PbTe, SnSe, and NaCo_2_O_4_-based thermoelectric fiber. In addition to the synthesis of electrospinning, two-step anodic oxidation process, and dissolved phase deposition, etc., it also introduces the thermal drawing method to fabricate some excellent performance fiber-based materials, which are detailed by continuously pulling microscopic fibers from a macroscopic preform in a controllable manner [21,22,23,24]. Moreover, the corresponding advanced characterization, performance evaluation, and the potential application of wearable thermoelectric fabrics or devices in power generation are summarized in this work.

## 2. Inorganic Synthetic Fibers

Inorganic fiber-based thermoelectrics exhibit more excellent thermoelectric performance and designable nanostructures, compared with organic or inorganic/organic hybrid, which can be attributed to their suitable electronic structures and low lattice thermal conductivity. Thus, various types of low-dimensional thermoelectric materials such as nano- or micro-based fibers have been applied in miniatured thermoelectric devices or flexible thermoelectric generators.

### 2.1. Bi_2_(Te, Se)_3_-Based Nanofibers

**Figure 1:**
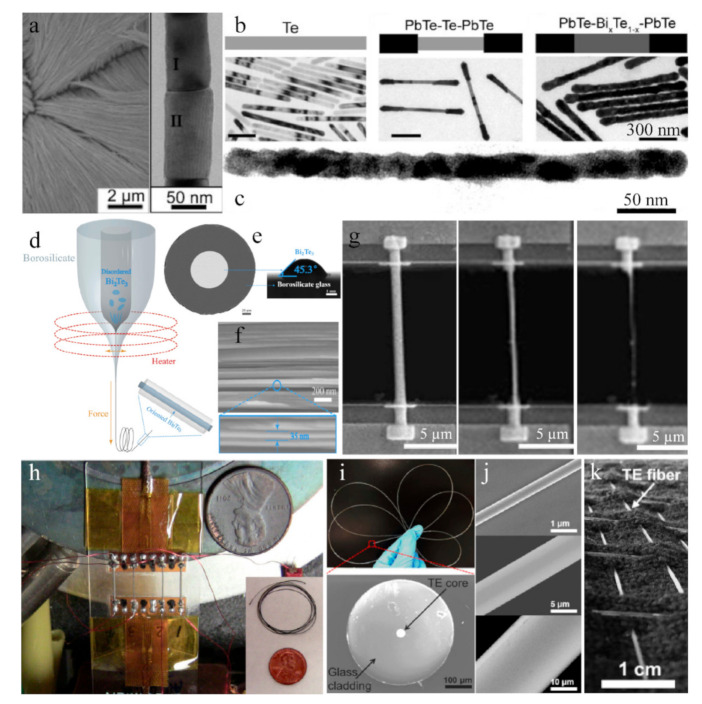
(**a**) Scanning electron microscopy (SEM) images of the Bi_2_Te_3_/Te multisegmented nanowires based on the top-view and close-up observation. Reproduced with permission [26]. Copyright 2007, American Chemical Society. (**b**) Schematic sketch and microstructure characterization for the formation of PbTe/Bi_x_Te_1–x_/PbTe dumbbell-like, heterostructured nanowires. Reproduced with permission [27]. Copyright 2010, Wiley. (**c**) TEM images of the interfaces of an individual Te/Bi core/shell nanowire. Reproduced with permission [33]. Copyright 2008, Wiley. (**d**) Sketch of the molten core drawing process. (**e**) Electron micrograph image of the cross-section and (**f**) longitudinal section of the Bi_2_Te_3_ core fiber sample. Reproduced with permission [28]. Copyright 2018, American Institute of Physics. (**g**) The SEM images of pristine and trimmed Bi–Sb–Te nanowire with sizes of 750, 490, and 285 nm, respectively. Reproduced under a Creative Commons Attribution 4.0 International License [29]. Copyright 2016, Springer Nature. (**h**) A miniature uni-couple fabricated from the sintered p-type/B_i0.4_Sb_1.6_Te_3_ and n-type/Bi_2_Te_2.7_S_e0.3_ fibers. Reproduced with permission [30]. Copyright 2016, Springer. (**i**) Single TE fiber and (**j**) its cross-sectional SEM image. (**k**) A large-area wearable TE device constructed by TE fibers. Reproduced with permission [32]. Copyright 2017, Elsevier.

The Bi–Sb–Te alloy system has obtained extensive attention due to its excellent thermoelectric performance near room temperature. The Bi_2_Te_3_/Te multiple heterostructured nanowire arrays fabricated via a two-step anodization process can be understood by the precipitation reaction of the supersaturated Bi_0.26_Te_0.74_ alloy under a nanoconfined system [25]. Figure 1a depicts the FE-SEM and TEM images of Bi_2_Te_3_/Te nanowire arrays annealed at 300 °C, and the close-up of a coupled segments can be well indexed to Te (segment I) and Bi_2_Te_3_ (segment II), respectively [26]. By regulating the reaction conditions, we can obtain some fiber-based products with a unique morphology. By injecting Bi precursor into the colloidal suspension of PbTe/Te/PbTe nanowires (Figure 1b), the dumbbell-like heterostructured PbTe/Bi_x_Te_1–x_/PbTe nanowires were prepared, which have different characteristics of the Te/Bi core/shell nanowire by using single-crystal Te nanowires (see Figure 1c) [27]. Moreover, using a special drawing technique of glass-cladding fibers, the continuous Bi_2_Te_3_-core fiber with a bend radius of less than 5 cm was obtained, as shown in Figure 1d. The fiber shows excellent continuity and uniformity feature, with the observed wetting angle at 45.3° (see Figure 1e), indicating that the wetting property of the two materials is superior at drawing temperature ~700 °C. The SEM image for the longitudinal section of the Bi_2_Te_3_ fibers is visualized horizontally in Figure 1f, which is parallel to the fiber symmetry axis and reveals many alternately dark and bright parallel stripes with an average interval of 30~40 nm (blue circle region). Such characteristics have suggested a nanosheet structure of Bi_2_Te_3_ fibers [28]. Another fiber synthesis process was idealized by the focused ion beam (FIB) technique, where a single nanowire was trimmed down from 750 to 285 nm by FIB, shown in Figure 1g, such as Bi-Sb-Te nanowires. Such in situ techniques have various advantages to control the reduction of nanowires size, and measurement of intrinsic thermoelectric properties to avoid the easier oxidation of vast surfaces in air [29]. In general, these nanowire or nanofiber structured materials ultimately need to be designed with an appropriate size to possess a certain strength and flexibility. Prototypes of miniatured modules consisting of up to four uni-couples composed of p-type Bi_0.5_Sb_1.5_Te_3_ and n-type Bi_2_Te_2.7_Se_0.3_ sintered fibers are displayed in Figure 1h. As a result, the as-extruded fibers were very flexible and could be formed into various shapes, which can be used in various applications of portable and wearable devices (inset of Figure 1h) [30]. Moreover, a single bendable thermoelectric fiber was formed from the nanoscale to microscale (Figure 1j) through thermal drawing process [31], which was specified by continuously pulling microscopic fibers from a macroscopic preform in a controllable manner; this technique usually requires that the Tg (glass transition temperature) of the cladding materials needs to be higher than the Tm (melting point) of the functional core materials. Through regulating the preform feeding and the fiber drawing speed, the fiber core diameter can be controlled within a wide range (from tens of nanometers to several millimeters) and the built-in stress between the cladding and core materials induced by the mismatch of thermal expansion coefficients can be optimized. The SEM image of cross-section indicates that the fiber possesses excellent flexibility and a clean interface between the thermoelectric core and the glass cladding (Figure 1i). Furthermore, those p-type Bi_0.5_Sb_1.5_Te_3_ and n-type Bi_2_Se_3_ TE fibers were constructed in a wearable fabric for flexible electronics on large areas (Figure 1k) [32].

### 2.2. PbTe-Based Fibers

**Figure 2:**
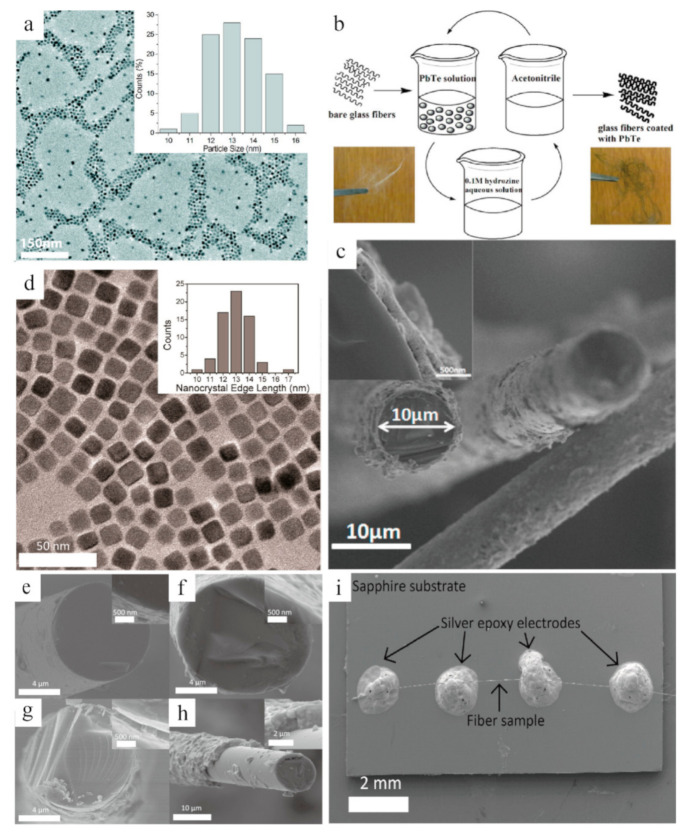
(**a**) TEM image of PbTe nanocrystals; upper inset is the size distribution of PbTe nanocrystals. (**b**) Scheme of the coating procedure to fabricate the bare glass fibers and PbTe nanocrystal-coated glass fibers. (**c**) SEM image of glass fibers coated by PbTe nanocrystal with a thickness ~300 nm. Reproduced with permission [34]. Copyright 2012, American Chemical Society. (**d**) Low-resolution TEM image of PbTe nanocrystals with the inset of size distribution. (**e**–**h**) Cross-section SEM images of cut ends of glass fibers based on PbTe samples. (**i**) SEM image of the PbTe fiber sample used for 3ω measurements. Reproduced with permission [38]. Copyright 2013, American Chemical Society.

Thermoelectric materials coated by the nanocrystals can be combined with the glass fibers, which can be mass-produced in an unlimited length and induce a lower thermal conductivity. For example, flexible glass fibers were coated with PbTe nanocrystals via a scalable solution-phase deposition method [34]. From the low-resolution TEM image of Figure 2a, it can be easily seen that the PbTe uniform nanocrystals were dispersed in an average size around 10 nm. The solution-phase deposition process (Figure 2b) consists of the following procedures: by dipping bare fluffy glass fibers into PbTe nanocrystal solution, removing the capping ligands of its surface, and discarding hydrazine with nitrogen [35,36,37]. The coated glass fibers have a uniform diameter of 10 um with 300 nm of PbTe nanocrystal coating (Figure 2c), while the cubic nanocrystals are distributed with an edge length of 12.9 ± 1.1 nm, presented in Figure 2d. Moreover, to obtain the thermoelectric parameters of glass fibers coated with PbTe nanocrystals, a single fiber with self-heated 3ω technique can be understood from Figure 2i [38,39]. On the other hand, the samples with various nanocrystal (having various coating thicknesses produced by varying coating cycles) are displayed in the cross-section of Figure 2e–h. Based on these synthesized glass fibers and measurement platforms, the experimental results suggest that the glass fibers with a small volume fraction of PbTe nanocrystals lead to an increased thermal conductivity and reduce with a higher volume fraction. These results are useful for optimizing the thermal measurement for some fiber- or wire-shaped samples with low thermal conductivity [38], which thereby further promote the applications of the 3ω measurement technique in fiber- or wire-shaped thermoelectrics.

### 2.3. Ag_2_Te-Based Fibers

**Figure 3:**
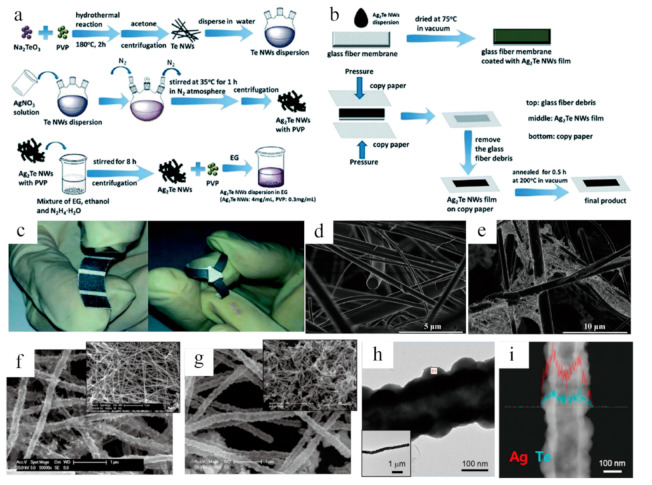
(**a**) Schematic diagram demonstrating Ag_2_Te nanowires and (**b**) the detailed process of fabricating paper coated with Ag_2_Te films. (**c**) One piece of paper constructed by four sheets of parallel (9 mm × 5 mm) Ag_2_Te nanowire films and a clover-like shape with three sheets (20 mm × 3 mm). (**d**) Representative SEM images of Ag_2_Te glass fiber sheet and (**e**) coated with Ag_2_Te nanowires dispersions. Reproduced with permission from Royal Society of Chemistry [41]. (**f**–**g**) High-definition SEM images of synthesized Ag_x_Te_y_ hollow nanofibers; inserted are low-magnification pictures. (**h**) The TEM pictures and (**i**) line-scan EDS analysis of Ag_x_Te_y_ nanofibers. Reproduced with permission [40]. Copyright 2015, American Chemical Society.

It is known that the flexibility of inorganic semiconductors is too difficult to transfer them into bendable or foldable films by themselves. Some flexible substrates, such as polyvinylidene fluoride (PVDF) and glass fiber, are necessary to support the inorganic films, which have emerged as a novel flexible substrate of thermoelectric films owing to their features of lightness, foldability, and breathability [34,40]. Miao et al. reported a novel glass-fiber-aided cold-press method for achieving flexible n-type Ag_2_Te films on a copy-paper substrate [41]. As shown in Figure 3a, the dispersion of Te nanowires (NWs) was firstly obtained through a hydrothermal reaction [42] and then by pouring AgNO_3_ solution into Te NWs to form Ag_2_Te NWs. After removing the surface ligands under vigorous stirring, the pure Ag_2_Te NWs were finally collected by centrifugation and washing process. Based on the synthesis of Ag_2_Te NWs, the corresponding dispersion droplets were dried on a glass fiber sheet and then sandwiched between copy papers. After pressing at a certain pressure, the Ag_2_Te NWs film was tightly adhered to the surface of the copy papers (Figure 3b). For better understanding, four sheets of parallel Ag_2_Te NWs films on a paper substrate and three sheets of films with a clover-like shape were selected, as shown in Figure 3c. The SEM images (Figure 3d,e) of the formed film reveal a porous structure of interlaced glass fiber sheet, and the substrate is hardly covered by the Ag_2_Te NWs. Different from the abovementioned method, another technique of synthesizing ultra-long Ag_x_Te_y_ nanofibers was carried out via combining the electrospinning with galvanic displacement reaction (GDR), by putting electrospun Ni nanofibers [43] into TeO_2_, AgNO_3,_ and HNO_3_ solution at room temperature. Thus, the branched and continuous Ag_x_Te_y_ nanofibers facilitate the regulation of concentrated Ag^+^ for electrolytes. As shown in Figure 3f, a low Ag^+^ concentration of 0.01 mM was beneficial to obtain the nanofibers with similar morphology to the pristine Te nanofibers [44]. Conversely, the growth of branched structures (see Figure 3g) was observed with higher Ag^+^ concentrations (i.e., 0.2 mM), leading to the preferential deposition in the (0 0 1) direction, which may be attributed to the limited mass transfer of HTeO_2_^+^ ions as clarified by Zhang et al. [40]. The bright-field TEM analysis of an as-formed nanofiber (Figure 3h) shows a nodular surface and hollow structure, and the line-scan EDS (Figure 3i) further reveals that the hollow structure was composited by a nominal Ag_x_Te_y_.

### 2.4. SnSe-Based Fibers

**Figure 4:**
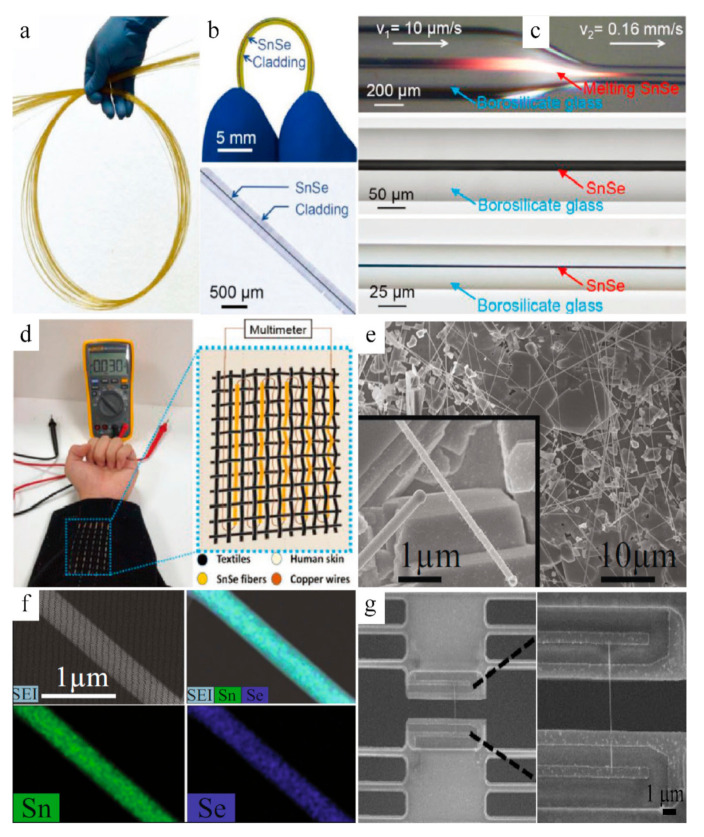
(**a**) Photograph of SnSe fibers. (**b**) Single SnSe fiber and its optical microscope image without polymer coating. (**c**) Fabrication of single-crystal SnSe-core fiber based on CO_2_ laser taper process. (**d**) Demonstrations of multidimensional SnSe fabric for converting heat to electricity. Reproduced with permission [31]. Copyright 2020, Wiley. (**e**) Scanning electron microscopy (SEM) image of the as-grown SnSe nanowires. (**f**) Energy-dispersive X-ray spectroscopy mapping for a measured SnSe nanowire (480 nm diameter). (**g**) The low- and high-magnification SEM images of SnSe nanowire, from left to right, transferred onto the microdevice. Reproduced under a Creative Commons Attribution 4.0 International License [50]. Copyright 2018, Springer Nature.

The rigid single-crystal SnSe is the best ever with a high TE performance of 2.8; however, it is difficult to apply in flexible and wearable devices currently. With the help of the thermal drawing process, an ultralong single-crystal SnSe wire with a diameter from micro- to the nanoscale has been achieved by Zhang et al. The single-crystal SnSe fiber is polycrystalline, highly flexible, ultralong, and mechanically stable, as shown in Figure 4a, b. Flexible SnSe fiber with variable diameters can be achieved by the CO_2_ laser taper process without changing its single-crystal features (Figure 4c). Such fabrics woven by functional SnSe fibers enable the breathability of covered areas when placed on the human body (Figure 4d). For example, a shirt composed of flexible thermoelectric SnSe fibers can induce an output voltage of 30 mV accompanied by a temperature difference between skin and environment, due to the Seebeck effect. Commonly, miniatured thermoelectric devices require nanoscale materials. As for SnSe nanowires, the SEM image (Figure 4e) of the deposited nanowires fabricated by a catalyst-assisted thermal VLS (vapor–liquid–solid) process [45] reveals that SnSe nanowires were formed uniformly on the surface of the substrates with different diameters and lengths. Such NWs are also consistent with the X-ray spectroscopy (EDS) quantified results of Sn:Se with an atomic ratio~1:1 (Figure 4f). Such nanowires or nanofibers are usually for detaching from substrates and transporting to microdevices (FIB system) for thermoelectric properties measurement, as shown in Figure 4g [46,47,48,49].

### 2.5. NaCo_2_O_4_-Based Fibers

**Figure 5:**
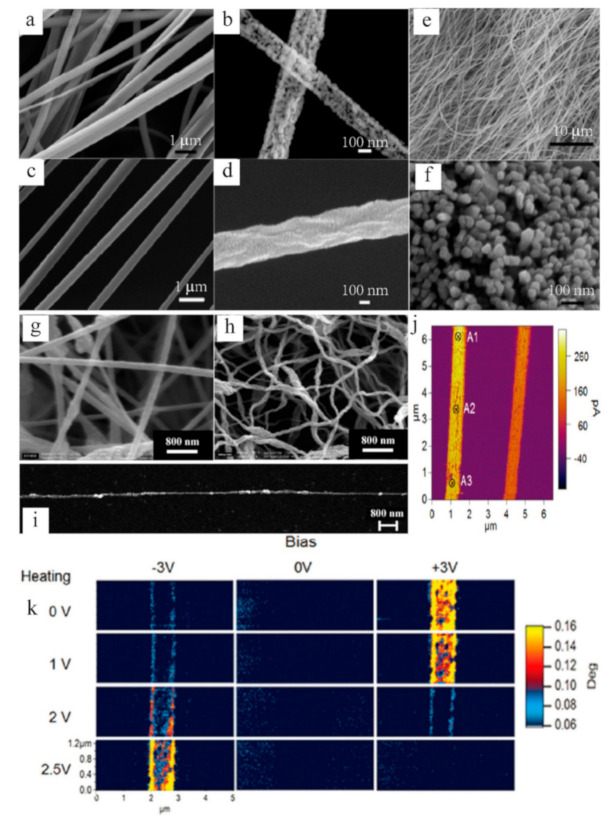
(**a**–**d**) SEM images of synthesized NaCo_2_O_4_ nanofibers using two different solvents before and after annealing. (**d**) Sketch of the molten core drawing process. (**e**) SEM image of as-spun nanofiber mat before annealing. (**f**) SEM images of nanofibrous mat annealed at 750 °C for 10 min. Reproduced with permission [61]. Copyright 2010, American Chemical Society. (**g**) SEM micrographs of sodium acetate/cobalt acetate/PAN composite fibers and (**h**) NaCo_2_O_4_ fibers. (**i**) A single-aligned NaCo_2_O_4_ fiber coated on Si substrate. Reproduced with permission [57]. Copyright 2006, Elsevier. (**j**) The measurement of I–V curve for nanofiber. (**k**) Electrostatic force microscopy (EFM) contrast variation in NaCo_2_O_4_ nanofiber vs. bias voltage with different heating voltages. Reproduced with permission [61]. Copyright 2010, American Chemical Society.

NaCo_2_O_4_, one of the potential oxide thermoelectric materials which carries a variety of attractive features of nontoxic and good chemical stability in the air or high temperature, possesses a layered crystalline structure similar to Ca_3_Co_4_O_9_ and Bi_2_Sr_2_Co_2_O_x_ [51,52,53,54]. Recent reports reveal ZT values of 1.2 and 0.8 for single-crystal NaCo_2_O_4_ and polycrystalline NaCo_2_O_4_. In addition, literature reveals that polymer or ceramic fibers have been prepared by the convenient electrospinning. The lengths and diameters of internal fibers prepared by this approach can be achieved with a few microns to tens of nanometers through controlling the types of polymers and synthesis conditions [55,56]. Drawing support from a sol–gel-based electrospinning technique [57], the thermoelectric nanofibers with grainsize as small as 10 nm have been achieved in the NaCo_2_O_4_ system, which can extend the applications of flexible thermoelectrics. As shown in Figure 5a–d, different solvents of methanol and water treated with annealing lead to distinctive morphology of NaCo_2_O_4_ nanofibers. The nanofibers in Figure 5a,c show the continuous fibrous morphology with smooth surface before annealing, and the surfaces of nanofibers become rough and porous after calcination due to the volatile polyvinylpyrrolidone (PVP) polymers (Figure 5b,d). To further verify this influence of temperature, the SEM images of as-spun mat without annealing, and another one with 750 °C heating for 10 min, are displayed in Figure 5e,f, respectively. Such results demonstrate that it is important to control the calcination time for three-dimensional growth mode of grains only. Interestingly, another vivid case of synthetic NaCo_2_O_4_ nanofibers is built on the calcination of electrospinning CH_3_COONa/Co(CH_3_COO)_2_/PAN precursor mixture, indicating an opposite effect of temperature on nanofiber growth. As shown in Figure 5g,h, compared with the original composite nanofibers (CH_3_COONa/Co(CH_3_COO)_2_/PAN) in Figure 5g, the diameters of NaCo_2_O_4_ nanofibers became even smaller about 30–80 nm after annealing at 400 °C (Figure 5h). Furthermore, by increasing the calcination time to 5 h, a single aligned NaCo_2_O_4_ nanofiber collected over a gap formed between two strips of silicon substrate [58] exhibits the long-range orderly filamentous characteristic (Figure 5i). The corresponding electrical and thermal transport properties of the NaCo_2_O_4_-based fiber system can be measured by the local I–V curve on three spots of the nanofiber. However, the difference in color contrast reflects the disparity of overall electrical conductivity for NaCo_2_O_4_ fiber (see Figure 5j). Meanwhile, the electrostatic force microscopy (EFM) mapping equipped with a thermal probe [59,60] could display the changes at surface potential (electrical conductivity) of the fiber under different heating voltages (temperature gradient), as shown in Figure 5k. Such a phenomenon helps us to better observe the thermoelectric performance of a single fiber.

## 3. Breakdown and Conclusions

In this work, we reviewed the development of several classic inorganic thermoelectric fibers, which covered the Bi_2_(Te, Se)_3_- and Ag_2_Te-based thermoelectric fibers near room temperature, PbTe-based and SnSe-based thermoelectric fibers at middle temperature, and NaCo_2_O_4_–based thermoelectric fiber at high temperature. This article emphasizes that the micro- or nanofibers corresponding to different systems have their own intrinsic fiber morphologies, observed in the detailed SEM and TEM characterizations such as layered Bi_2_Te_3_, SnSe nanosheets, and cubic structure of PbTe. In addition, a single nanofiber or coated nanofibers with continuous and good roundness could be obtained by several advanced fiber prepared techniques. Specifically, the convenient electrospinning method was implemented to prepare the target fiber product with some auxiliary solvents in Ag_x_Te_y_ and NaCo_2_O_4_ fibers. Both the two-step anodization process and solution-phase deposition method are promising to obtain the segmented fibers and coated glass fibers due to their scalable and multifunctional feature. Unlike the FIB or self-heated 3ω technique, to achieve a single nanowire and to measure their thermoelectric properties simultaneously, thermal drawing based on glass-cladding fibers can produce large-scale and high-quality sub-microfibers. Such kinds of fibers exhibit flexible, bendable, and wearable charm for fabrics and portable electronics. The updated development of FTE materials is summarized with a detailed comparison in Table 1; thus, understanding this rapid development and advanced synthesis of fiber-based inorganic thermoelectrics is beneficial to solving the current challenges. In addition, we believe these thermoelectric fibers could play a greater role in the future of wearable and smart electronics.

## 4. Prospects and Future Development

Generally, inorganic thermoelectric fibers are promising because of their intrinsic flexibility, better performance, and varied regulations compared with organic fibers. However, their disadvantages, such as high cost and poor flexibility, limit their practical applications in wearable or portable thermoelectrics. Therefore, organic/inorganic hybrid fiber-based thermoelectrics may be an advantageous hotspot in future research of thermoelectric fibers. So far, it is challenging for the researchers to effectively balance the flexibility and performance of fiber-based materials. The abovementioned challenges require development of a new fabrication and processing technology that can optimize the thermoelectric properties such as nanostructuring and low-dimensional band structure, as well as introducing good flexibility and high continuity of the fibers. As for the applications of thermoelectric fibers, the heat conversions in wearable devices or fabrics need to be focused on in future research. For these materials, it is urgently required to realize their low cost, stability, flexibility, and durability. Besides, multifunctional materials integrating thermoelectric fibers with other functions such as biosensing fibers and optical fibers are favorable to develop a comprehensive energy conversion system. Referring to the advanced energy systems, there will be many obstacles and challenges in the commercialization of fiber-based thermoelectrics, but their promising applications in wearable and smart sensing areas is also fascinating.

## Figures and Tables

**Table 1:** A comparison of thermoelectric properties/device performance between different shapes (fibers, film, nanorods, fabrics, etc.) and various techniques detailed by inorganic/organic hybrid fibers, semiconductor fibers or coating on glass/silica fiber, and modified natural or manmade fiber/fabric of thermoelectric materials.

Material	Thermoelectric Properties			Device Performance			Reference
	S^2^σ (μWm^−1^K^−2^)	κ(Wm^−1^K^−1^)	ZT	Coupled Material	Output Voltage (mV)	Output Power (μW)	
**Inorganic/Carbon or Inorganic/Organic Hybrid Fiber-based Materials**							
rGO + Bi_2_Te_3_ films	108	-	0.0035	SWCNTs + Sb_2_Te_3_ films	67.5	23.6	[62]
SWCNT/ MoS_2_ buckypapers	52	6.8	0.0028	-	-	-	[63]
Carbon fibers + epoxy	245	-	-	-	19.56	0.87	[64]
CNTs/ PEDOT:PSS composite fibers	113	-	-	treated with hydrazine	8	0.43	[65]
SWCNTs/ PANI	217	0.44	0.15	-	8	~4	[66]
SWCNTs/ PVDF pastes	~378	-	-	doped by PEI	16	0.8	[67]
Cu_2_Se NWs/ PEDOT:PSS	270.3	0.25–0.3	0.3	-	15	0.32	[68]
Ta_4_SiTe_4_ whiskers + PVDF	1045.7	-	-	-	35	1.7	[69]
Te NWs + PEDOT:PSS fibers	78.1	-	-	coated by Ag	25.9	0.2	[70]
Te nanorods + SWCNTs + PANI	101	0.3	0.101	-	8	1	[71]
Te nanorods coated by SWCNT/PEDOT:PSS	104	-	-	treated with H_2_SO_4_	5.6	0.0536	[72]
**Semiconductor Fiber-based Materials or Coating on Glass/Silica Fiber**							
Ag_2_Te NW films	359.76	-	-	-	3.6	-	[73]
Bi_0.5_Sb_1.5_Te_3_ fibers	170–260	-	-	Bi_2_Te_2.7_ Se_0.3_ fibers	4.8	0.018	[30]
Si NWs	-	-	-	-	27.9	0.47	[74]
Si nanotube fabrics	-	-	0.34	-	22	-	[75]
Bi_0.5_Sb_1.5_Te_3_ core fibers	3529	0.84	1.25	Bi_2_Se_3_ core fibers	97	-	[32]
Ni-Ag coated silica fibers	-	-	-	-	0.9	0.002	[76]
PbTe nanocrystalcoated glass fibers	406	0.226	0.75	-	-	-	[34]
**Modified Natural or Man-made Fiber/Fabric**							
Cellulose fibers coated by PEDOT:PSS	1.5	0.1	0.0013	Ni foils	6	2.4	[77]
Cellulose fibers + SWCNTnetworks	8.1	-	-	treated with PEI	~16.8	0.0755	[78]
Cellulose fibers + Bi_2_Te_3_	377.5	0.47	0.38	Cellulose fibers + (Bi,Sb)_2_Te_3_	144	-	[79]
Glass fabric printed by Bi_2_Te_3_	1029.3	0.93	0.33	Glass fabric printed by Sb_2_Te_3_	90	-	[80]
Glass fabric screen-printed by Bi_2_Te_2.7_Se_0.3_	2077.3	0.37	0.81	Glass fabric screen-printed by Bi_0.5_Sb_1.5_Te_3_	-	-	[81]
Nylon membrane with Ag_2_Se films	~987.4	0.478	0.6	-	18	0.46	[9]
Polymer fabrics printed by Bi_0.5_Sb_1.5_Te_3_	-	-	-	Polymer fabricsprinted by Bi_2_Se_0.3_Te_2.7_	25	0.24	[82]
Silk fabric deposited by Sb_2_Te_3_	-	-	-	Silk fabric deposited by Bi_2_Te_3_	~10	~0.015	[83]

## Data Availability

Not applicable.
